# Chemical Fingerprint Analysis and Ultra-Performance Liquid Chromatography Quadrupole Time-of-Flight Mass Spectrometry-Based Metabolomics Study of the Protective Effect of Buxue Yimu Granule in Medical-Induced Incomplete Abortion Rats

**DOI:** 10.3389/fphar.2020.578217

**Published:** 2020-11-30

**Authors:** Yan Zhang, Wei Li, Ting-Ting Chen, Yong Yang, Meng-Yao Wu, Jie-Ying Luo, Yun Gong, Liang Zou

**Affiliations:** ^1^ School of Medicine, Chengdu University, Chengdu, China; ^2^ Department of Pharmacology, Zhuzhou Qianjin Pharmaceutical Co., Ltd., Zhuzhou, China; ^3^ College of Pharmacy, Hunan University of Chinese Medicine, Changsha, China; ^4^ Key Laboratory of Coarse Cereal Processing of Ministry of Agriculture and Rural Affairs, School of Food and Biological Engineering, Chengdu University, Chengdu, China

**Keywords:** Buxue Yimu granule, medical abortion, anti-inflammatory, ultra-performance liquid chromatography quadrupole time-of-flight mass spectrometry, metabolomic, fingerprint analysis

## Abstract

Medical abortion is a common method to terminate an early pregnancy and often causes serious complications such as abnormal uterine bleeding and endometritis. Buxue Yimu granule (BYG) is a well-known traditional Chinese medicine prescription composed of five kinds of drugs and is widely used in gynecology and obstetrics. The aim of the present study was to establish the quality standard of BYG and investigate its protective effect on incomplete abortion. The chemical fingerprint of BYG was established by high performance liquid chromatography (HPLC). The major compounds of BYG were determined by ultra-performance liquid chromatography with triple quadrupole mass spectrometry. An incomplete abortion rat model was induced by intragastric administration of mifepristone (8.3 mg·kg^−1^) combined with misoprostol (100.0 μg·kg^−1^) during early pregnancy. The serum levels of human chorionic gonadotrophin (HCG), estradiol (E_2_), and progesterone (PG) were determined. The serum endogenous metabolites were analyzed by ultra-performance liquid chromatography quadrupole time-of-flight mass spectrometry (UPLC-Q-TOF/MS). Multivariate analysis, including partial least squares discriminant analysis (PLS-DA) and orthogonal partial least squares discriminant analysis (OPLS-DA), was employed to analyze the metabolic profiles, and MetaboAnalyst was used to investigate the metabolic pathways. Furthermore, hematoxylin-eosin staining (HE) was used to evaluate the histopathological changes in uterine tissue. The expression levels of VEGFA and NF-κB were detected by immunohistochemistry. The results indicated that HPLC fingerprint analysis can be successfully used to assess the quality of BYG. The medical-induced incomplete abortion rats were clearly separated from control rats, and the biochemical changes were gradually restored to normal after administration of BYG. Moreover, 19 potential biomarkers, including N-lactoylleucine, 2-piperidinone, isobutyryl-l-carnitine, eicosapentaenoylcholine, LysoPC(14:0), LysoPC(20:5), physagulin C, LysoPC(18:3), leukotriene D5, deoxycholic acid 3-glucuronide, glycine, pregnanediol 3-O-glucuronide, LysoPC(18:2), LysoPC(17:0/0:0), N-acetyl-leukotriene E4, LysoPC(18:0), platelet-activating factor, LysoPA(24:1), and LysoPC(18:1), which were mainly related to the amino acids metabolism, lipids metabolism, and bile acid biosynthesis, were identified. Consequently, BYG exerts a potential protective role in the intervention of incomplete abortion by anti-inflammatory, promote endometrial repair, and regulate the metabolic disorders.

## Introduction

The availability of mifepristone with misoprostol for medical abortion has been increasingly used as an alternative method to surgical intervention for early termination of pregnancy (Baiju et al., 2019). In the United States, the Food and Drug Administration (FDA) has approved this combination of medications for use at home at up to 10 weeks’ gestation (Schmidt-Hansen et al., 2020). Most women believe that medical abortion has higher autonomy than surgical abortion and is more convenient, can protect privacy, and has fewer side effects than surgery (LaRoche and Foster, 2020). Despite excellent effectiveness and safety, however, previous studies have shown that the drug may still cause incomplete abortion, with a complete successful expulsion rate of 92.3% for 64–70 days of pregnancy and 86.7% for 71–77 days of pregnancy (Dzuba et al., 2020). Histopathological examination showed that the residual embryo of incomplete abortion may be the cause of other severe side-effects such as abnormal uterine bleeding (AUB), pain, and pelvic infection (Hsia et al., 2019).

From the perspective of Western medicine, uterine surgery is required for patients with an incomplete abortion. At the same time, for the treatment of complications of medical abortion, such as uterine contraction, hemostasis, and anti-infection treatments, are mainly used in the clinic (Chen and Creinin, 2015). From the perspective of traditional Chinese medicine, most women have the characteristics of weakness of Qi and blood and stagnation of blood stasis after childbirth or induced abortion. Buxue Yimu granule (BYG) is a famous prescription composed of *Leonurus japonicus* Houtt. (Yimucao), *Angelica sinensis* (Oliv.) Diels (Danggui), *Astragalus membranaceus* Bunge (Huangqi), *Citrus Reticulata* Blanco (Chenpi), and Colla corii asini (Ejiao), which has the effect of replenishing Qi and blood, promoting circulation and mitigating blood stasis. Moreover, clinical evidence has shown that BYG has a definite curative effect in the treatment of postpartum or abortion complications. However, the biological mechanisms of BYG in the treatment of obstetric complications are unclear.

Metabolomics mainly studies the changes in endogenous molecule metabolites such as fatty acids, amino acids, lipids and organic acids caused by interferences from the environment, disease status, drug intervention and other factors (Liu et al., 2012). The research concept complements the holistic view in the system theory of traditional Chinese medicine. At present, metabolomics has been widely used in the fields of food safety (Liu and Wu, 2020a), disease diagnosis (Wang et al., 2020), and drug action mechanistic studies. In particular, this technique provides a good strategy for evaluating the biological mechanism of complex classical Chinese medicine prescriptions. For instance, ^1^H-NMR-based metabolomics has been used to study the mechanism of HuangQi-DanShen in cerebral ischemia (Liu et al., 2020b). A metabolomics study based on HPLC-MS/MS predicts that the treatment of hyperlipidemia by Si-miaoyong-an decoction is related to its antioxidant and anti-inflammatory activities and the regulation of pyruvate and taurine metabolism (Shi et al., 2020). Tianma Gouteng decoction exhibited the antihypertensive activity by regulating the metabolism of amino acids, sphingomyelin, and glycerol phospholipids (Dong et al., 2020).

In the present study, the quality standard of BYG was established by high-performance liquid chromatography (HPLC). The therapeutic effects of BYG on serum hormone levels and uterine histopathology were investigated in rats having incomplete abortions induced by mifepristone and misoprostol. Moreover, an ultra-performance liquid chromatography quadrupole time-of-flight mass spectrometry (UPLC-Q-TOF/MS)-based metabolomic method combined with multivariate analysis was applied to demonstrate and identify the potential biomarkers and the metabolic pathways.

## Materials and Methods

### Chemicals and Reagents

Buxue Yimu granule was obtained from Zhuzhou Qianjin Pharmaceutical Co., Ltd. (Hunan, China). Hesperidin (batch No. C-006-191012), Astragaloside (batch No. H-013-180228), leonurine (batch No. Y-065-180518), ferulic acid (batch No. A-002-181216), and calycosin (batch No. M-020-190219) were obtained from Chengdu Herbpurity Co., Ltd. Senkyunolide A (batch No. wkq 20050703) was purchased from Chengdu Weikeqi Biotechnology Co., Ltd. Ligustilide (batch No. DST 191015-007) and Formononetin (batch No. DST191202-011) were obtained from Chengdu Desite Biotechnology Co., Ltd. Mifepristone and Misoprostol were obtained from Zizhu Pharmaceutical Co. (Peking, China). Pg ELISA kits (batch No. HXPZ89W47A) and E2 ELISA kits (batch No. DCZXS4SYKL) were purchased from Elabscience Biotechnology Co., Ltd. (Wuhan, China). The HCG ELISA kit (batch No. 12/2019) was purchased from Shanghai MLBIO Biotechnology Co., Ltd. (Shanghai, China). Anti-VEGFA antibody (ab9570) (batch No. 20334270) was obtained from Abcam Company Ltd. (Cambridge, MA, United States). NF-κB antibody (batch No. 00085044) was obtained from Sanying Biotechnology Co., Ltd. (Wuhan, China) HPLC-grade methanol and acetonitrile were purchased from Thermo Fisher Scientific Inc. (Iowa, United States). Formic acid was purchased from Sigma Chemical Co. (St. Louis, MO, United States). The water used in this study was prepared by an ULUP ultrapure water purification system (Chengdu, China).

### Chemical Fingerprint Analysis of Buxue Yimu Granule

Buxue Yimu granules (3 g) was extracted by an ultrasonic extractor with 10 ml of water for 10 min, and then approximately 40 ml of acetonitrile was added and sonicated for 30 min. After cooling, acetonitrile was added to bring the volume to 50 ml, the solution was filtered through a 0.22 μm microporous filter membrane, and the subsequent filtrate was collected for analysis.

Chromatographic separation was carried out on an LC-16-PDA HPLC system (SHIMADZU, Japan) using a SymmetryShield™ RP C18 column (4.6 mm × 250 mm 5.0 µm, Waters, United States) with the column temperature maintained at 35°C and a detection wavelength of 254 nm. Formic acid (0.1%) in water and acetonitrile were treated as mobile phases A and B, respectively. Gradient elution was programmed as follows: 0–8 min, 95% A; 8–60 min, 95–10% A; 60–63 min, 10–2% A; 63–66 min, 2–95% A; and 66–72 min, 95% A. The sample injection volume was set at 10 µL for analysis with a flow rate of 1.0 ml·min^−1^.

Chromatographic data were processed by the SHIMADZU Labsolutions analysis workstation, and the similarity analysis was conducted according to the similarity evaluation system of the chromatographic fingerprint of Traditional Chinese medicine (2012.130723 version, Chinese Pharmacopoeia Committee).

### Determination of the Major Compounds in Buxue Yimu Granule

Chromatographic separation was carried out on a Vanquish UPLC system (Thermo Fisher Scientific, United States) using an Accucore™ C18 column (2.1 mm × 100 mm 2.6 µm, Thermo Fisher, United States) with the column temperature maintained at 35°C. Formic acid (0.1%) in water and acetonitrile were treated as mobile phases A and B, respectively. The gradient elution was programmed as follows: 0–5 min, 15–40% B; 5–10 min, 40–45% B; 10–18 min, 45–70% B; 18–18.01 min, 70–15% B and 18.01–23 min, 15% B. The sample injection volume was set at 10 µL for analysis with a flow rate of 0.2 ml·min^−1^.

Mass spectrometry was performed on a TSQ Fortis triple quadrupole mass spectrometer system (Thermo Fisher Scientific, United States) equipped with an electrospray ionization source operating in positive ion mode with the following parameters: scan type, SRM; type of ion source, H-ESI; sheath gas flow rate, 35 arb; aux gas flow rate, 15 arb; spray voltage, 3.5 kV, capillary temperature, 350°C; and aux gas heater temperature, 350°C.

### Medically Induced Incomplete Abortion Rat Model

Healthy, SPF-grade adult male and female Sprague-Dawley rats (180 g ± 20 g Certificate No. SCXK (Chuan) 2015-030) were supplied by the Animal Breeding Center of DaShuo Biotechnology Co., Ltd. (Chengdu, China). All rats were maintained on an alternating 12 h light/dark cycle, at the temperature of 22–25°C and humidity of 55–60%. The rats were fed with a standard diet and given free access to water. Experimental procedures were strictly in accordance with the Guide for the Care and Use of Laboratory Animals and were approved by the Animal Ethics Committee of Chengdu University.

Female SD rats in estrus were mated with male rats in a separate cage at a ratio of 2:1 overnight. Observation of sperm in vaginal secretion smears the next morning by an optical microscope was considered to be an evidence of pregnancy, and that day was recorded as the first day of gestation. On the seventh day of pregnancy, six pregnant rats were randomly selected as the control group, and the remaining rats were given mifepristone (8.3 mg·kg^−1^, 8:00 a.m.) and misoprostol (100.0 μg·kg^−1^, 6:00 p.m.) by intragastric administration to induce incomplete abortion (Camilleri et al., 2019; Li et al., 2020). A cotton ball of appropriate size was placed in the vagina and removed at 8:00 and 20:00 the next day. When vaginal bleeding was observed, the medical-induced incomplete abortion of early pregnancy rats was considered to be successfully replicated. The model rats were randomly divided into the model group and the BYG group (4.32 g·kg^−1^). The BYG group was given the corresponding drugs by intragastric administration, and the control group and the model group were given an equal volume of normal saline once a day for 7 days.

### Determination of Serum Human Chorionic Gonadotrophin, Estradiol and Progesterone Levels

Blood samples were collected from the abdominal aorta after the last treatment and centrifuged at 3,500 rpm and 4°C for 10 min. Serum was aspirated and stored at −80°C until analysis of human chorionic gonadotrophin (HCG), progesterone (Pg) and estradiol (E_2_) levels, which were determined by ELISA assay according to the manufacturer’s instructions.

### Histopathological Examination

Uterine tissues were immediately collected after the rats were sacrificed. The left uterus tissues were fixed in 4% paraformaldehyde solution, embedded in paraffin, sectioned serially into 5 µm sections (RM2235 microtome, Leica, Germany), and stained with hematoxylin and eosin (HE). The pathological changes in the endometrium were evaluated by three randomly selected visual fields at a magnification of ×200 using optical microscopy (CX31, OLYMPUS, Japan).

### Expression of Vascular Endothelial Growth Factor A and Nuclear Factor kappa-B by Immunohistochemistry

The uterine tissues were pretreated as previously described. The tissue sections (5 μm) were dewaxed with xylene for 25 min, and then treated with different concentrations of ethanol for hydration. The tissues were then incubated with 3% H_2_O_2_ deionized water for 10 min to inactivate the endogenous peroxidase activity. Then, tissues were rinsed again with PBS, normal goat serum blocking solution was added, and the mixtures were incubated at room temperature for 20 min. Then, the mixtures were incubated in primary antibody overnight at 4°C and incubated at 37°C for 1 h the next day. Then, the sections were rinsed three times for 5 min each with PBS, 100 μL of biotin-labeled II antibody was added, and the samples were kept at 37°C. After incubation for 1 h, the samples were washed for three times with PBS. The VEGFA or NF-κB proteins were stained by immersion in DAB for 10 min. The reaction was terminated by washing with tap water for 10 min. Anhydrous ethanol dehydration, xylene transparency, sealing and microscopic examination were performed. Three microscopic fields (200×) were selected for each slice, and the quantitative average optical density was analyzed by Image-Pro Plus image analysis system.

### Metabolomics Serum Sample Pretreatment

For UPLC-Q-TOF/MS analysis, 400 µL of acetonitrile was added dropwise to 100 µL of the serum sample for protein-precipitation and vortexed for 3 min. The mixture was centrifuged for 10 min at 12,000 rpm, and the supernatant was used for analysis.

### Ultra-Performance Liquid Chromatography Quadrupole Time-of-Flight Mass Spectrometry Condition

Metabolomics serum samples were analyzed by an UPLC-Q-TOF/MS system (Agilent, United States). An Acquity UPLC BEH C18 column (2.1 mm × 100 mm, 1.7 μm, Waters, United States) was used for separation. The gradient mobile phase was a mixture of 0.1% formic acid in water (A) and acetonitrile (B) with a flow rate of 0.35 ml·min^−1^ at 35°C. The linear gradient was as follows: 90–70% A (0–3 min) and 70–5% A (3–25 min).

The following MS parameters were employed: full scan range, 50–1,200 *m*/*z*; drying gas flow, 6 L·min^−1^; source drying gas temperature, 300°C; sheath gas temperature, 320°C, sheath gas flow, 12 L·min^−1^; nebulizer pressure, 1.0 bar; capillary voltage, 3.5 kV.

During the whole analytical process, quality control (QC) samples were analyzed every 10 samples. The stability of the analytical method was evaluated according to the relative standard deviation (RSD) of the retention time and intensity of 10 randomly selected characteristic ion peaks of QC samples.

### Data Analysis

Partial least-squares discriminant analysis (PLS-DA) and orthogonal partial least-squares discriminant analysis (OPLS-DA) were adopted for multivariate analysis by SIMCA. The potential biomarkers were identified with the following databases: https://hmdb.ca/, http://www.genome.jp/kegg/, http://metlin.scripps.edu/, http://www.lipidmaps.org/. All results were presented as the mean ± SD. Study data were analyzed using one-way analysis of variance (ANOVA) for significance comparison. Values of *p* < 0.05 were considered statistically significant.

## Results

### Chemical Fingerprint of Buxue Yimu Granule

**FIGURE 1:**
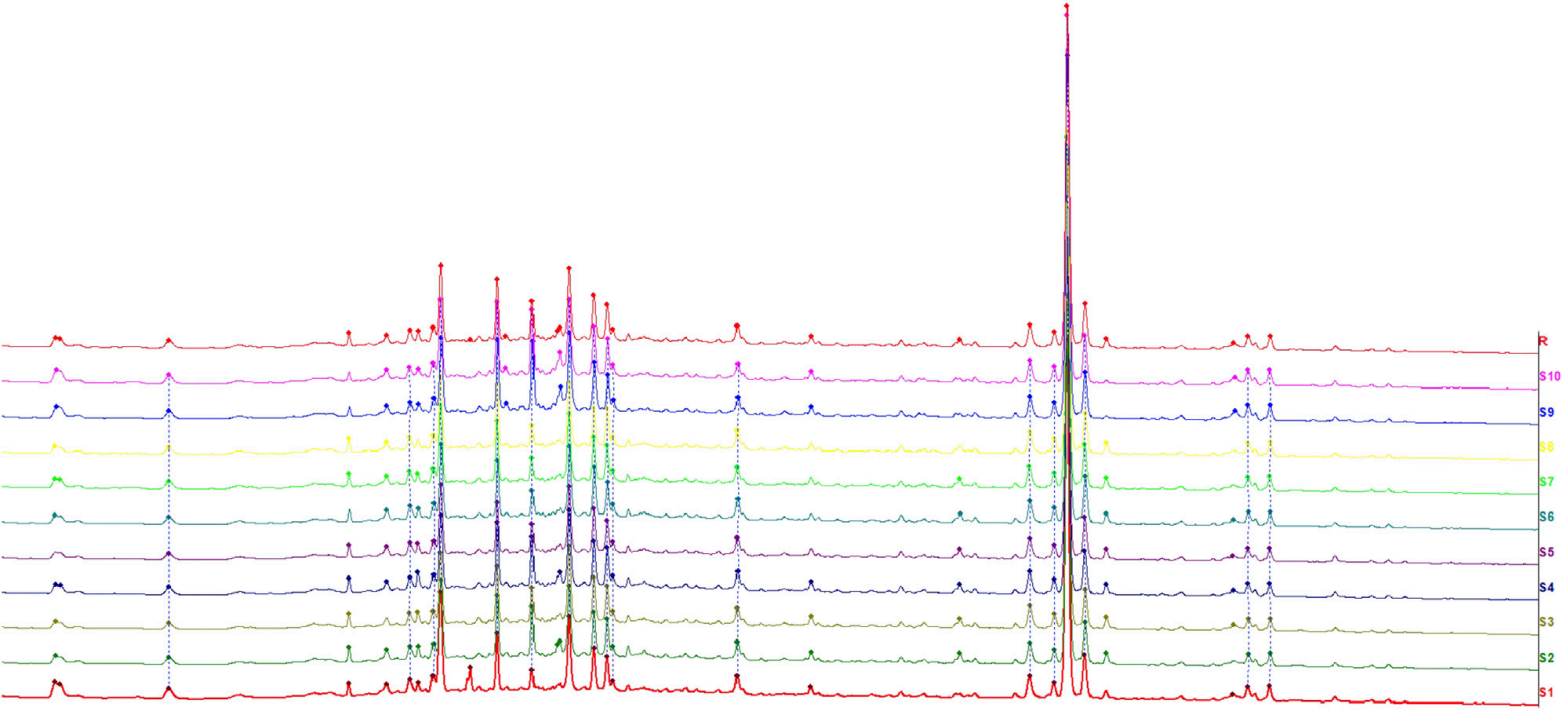
Representative HPLC chromatograms of BYG. R: reference substance S1–S10 were 10 batches of BYG.

Through the similarity comparison between the chromatograms of each sample, the common fingerprint chromatogram, namely, the reference chromatogram, was extracted, and 30 common fingerprint peaks were observed. Then the similarity of the chromatogram of each sample with the reference chromatogram was determined, and the similarity between each batch was greater than 0.99 (Figure 1). The experimental results showed that the similarity between the samples was high, and the preparation process was stable and controllable.

### Quantitative Analysis of Major Compounds in Buxue Yimu Granule

**FIGURE 2:**
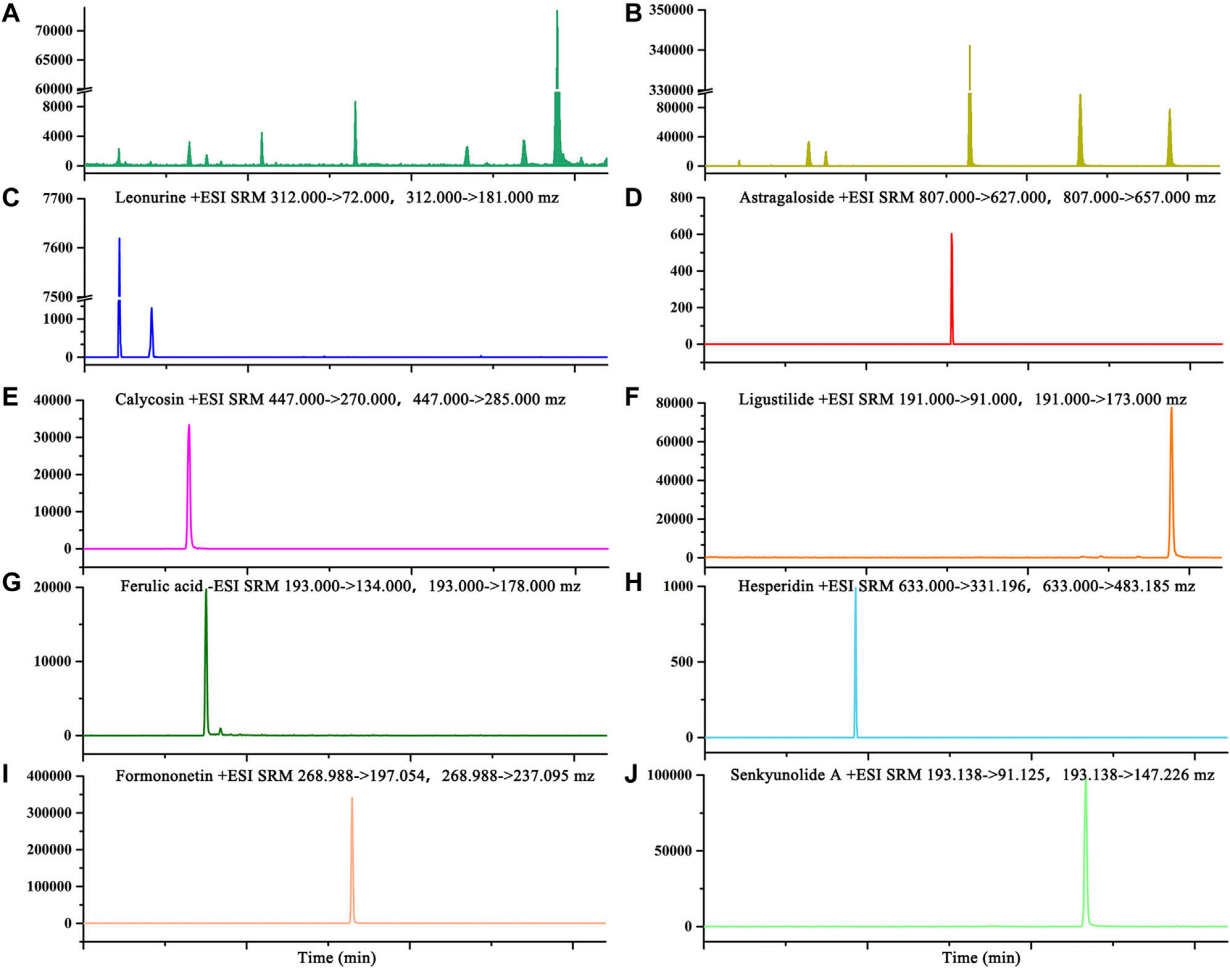
The typical MRM chromatograms of BYG **(A)**; mixed standard reference substance **(B)**; leonurine **(C)**; astragaloside **(D)**; calycosin **(E)**; ligustilide **(F)**; ferulic acid **(G)**; hesperidin **(H)**; formononetin **(I)**, and senkyunolide A **(J)**.

Seven major characteristic chemical constituents from BYG were determined. A typical MRM chromatogram was shown in Figure 2 and the mass spectrometric ion information of the major components was listed in Table 1. As a result, the concentrations of leonurine, calycosin, ferulic acid, hesperidin, formononetin, senkyunolide A, and ligustilide was 0.181 mg·g^−1^, 0.041 mg·g^−1^, 0.050 mg·g^−1^, 0.07 mg·g^−1^, 0.005 mg·g^−1^, 0.012 mg·g^−1^, and 0.385 mg·g^−1^, respectively. Astragaloside was not detected in Buxue Yimu granule extract.

**TABLE 1:** Mass spectrometry ion information of major components.

Compounds	Polarity	Precursor (*m*/*z*)	Product (*m*/*z*)	Collision energy (V)
Hesperidin	Positive	633	331.196	30.99
Hesperidin	Positive	633	483.185	31.75
Formononetin	Positive	268.988	197.054	36.63
Formononetin	Positive	268.988	237.095	26.9
Senkyunolide A	Positive	193.138	91.125	22.61
Senkyunolide A	Positive	193.138	147.226	9.34
Ligustilide	Positive	191	91	25.60
Ligustilide	Positive	191	173	15.03
Leonurine	Positive	312	72	26.86
Leonurine	Positive	312	181	21.51
Astragaloside	Positive	807	627	50.45
Astragaloside	Positive	807	657	46.70
Ferulic acid	Negative	193	134	13.47
Ferulic acid	Negative	193	178	11.53
Calycosin	Positive	447	270	38.91
Calycosin	Positive	447	285	15.57

### Effect of Buxue Yimu Granule on Serum Hormone Levels

**FIGURE 3:**
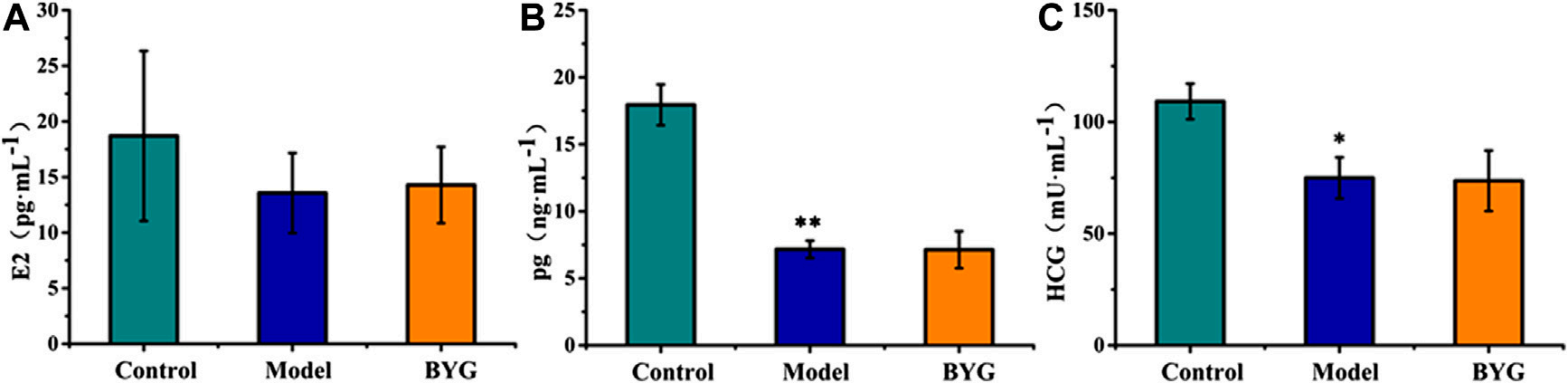
The level of E_2_ **(A)**, Pg **(B)**, and HCG **(C)** in medical-induced incomplete abortion rats. Note: VS control group, *p < 0.05.

As shown in Figure 3, compared with those in the pregnancy control group, the HCG, E_2_ and Pg levels in the model group were significantly decreased (*p* < 0.05). Compared with those in the model group, there was no significant difference between HCG, E_2_ and Pg levels in the BYG group. These results indicated that BYG had no significant effect on serum hormone levels at this dose.

### Effect of Buxue Yimu Granule on Pathological Endometrial Changes in Uterine Tissue

**FIGURE 4:**
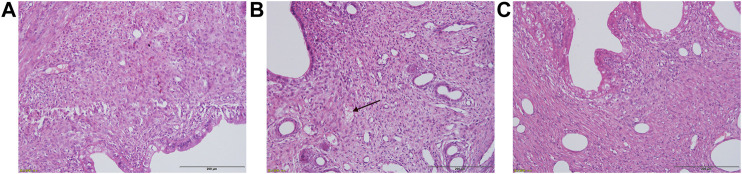
Representative histopathological micrographs of uterine tissue. **(A)** Pregnancy control group + saline solution treatment. **(B)** Model group + saline solution treatment. **(C)** Model group + BYG treatment. H&E stain, ×200.

As shown in Figure 4, the endometrium of the pregnancy group was intact. Congestion, edema and local endometrial defects were observed in some rats in the medical-induced incomplete abortion group. Compared with model rats, except for one case of abundant endometrial blood vessels and interstitial looseness in the BYG group, the rats showed no obvious abnormal changes. Thus, BYG could ameliorate the histopathological damage.

### Effect of Buxue Yimu Granule on the Expression of Vascular Endothelial Growth Factor A and Nuclear Factor kappa-B

**FIGURE 5:**
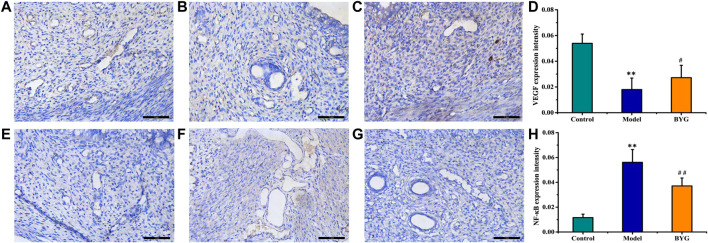
Immunohistochemistry images of VEGFA and NF-κB in the uterus tissue. X200.

As shown in Figure 5, the positive area of VEGFA was light yellow and mainly expressed in the cell membrane and cytoplasm, and the nucleus was blue. Compared with that in uterine tissue from the pregnancy control group, the expression of VEGFA in uterine tissue from the medical-induced incomplete abortion model group was decreased, and BYG treatment increased the expression of VEGFA. While the expression of NF-κB in uterine tissue from the medical-induced incomplete abortion model group was increased, and BYG treatment decreased the expression of NF-κB.

### Validation of Ultra-Performance Liquid Chromatography Quadrupole Time-of-Flight Mass Spectrometry Conditions

**FIGURE 6:**
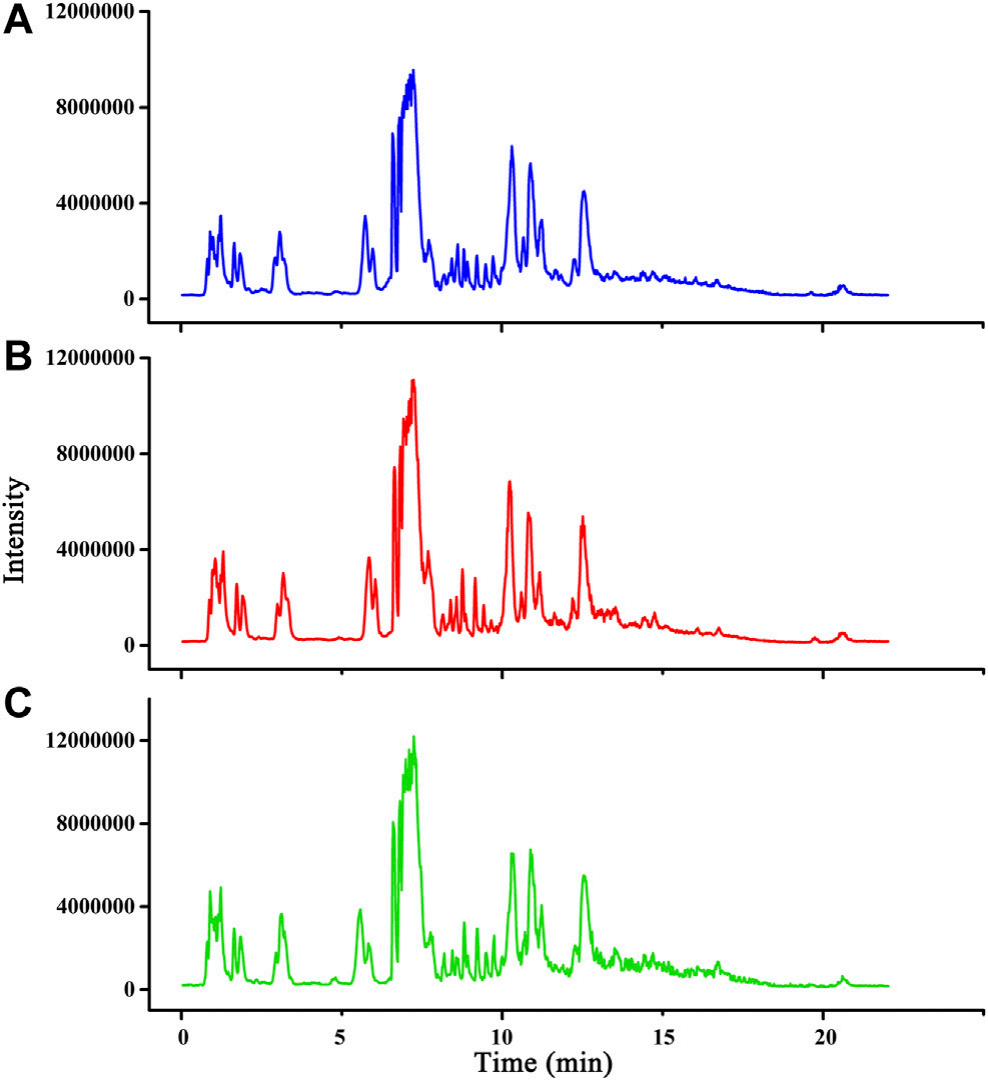
Representative UPLC-Q-TOF/MS chromatograms of the control (blue line), model (red line), BYG treatment (green line) serum samples.

The serum metabolite profiles were obtained from each rat group in positive ion mode. Representative UPLC-Q-TOF/MS chromatograms were presented in Figure 6. All the relative standard deviation (RSD %) values for the retention time and *m*/*z* data of the selected ions of QC sample were less than 8%, showing that the method established in this study for the analysis of rat serum metabolomics was accurate and stable.

### Identification of Potential Biomarkers

**FIGURE 7:**
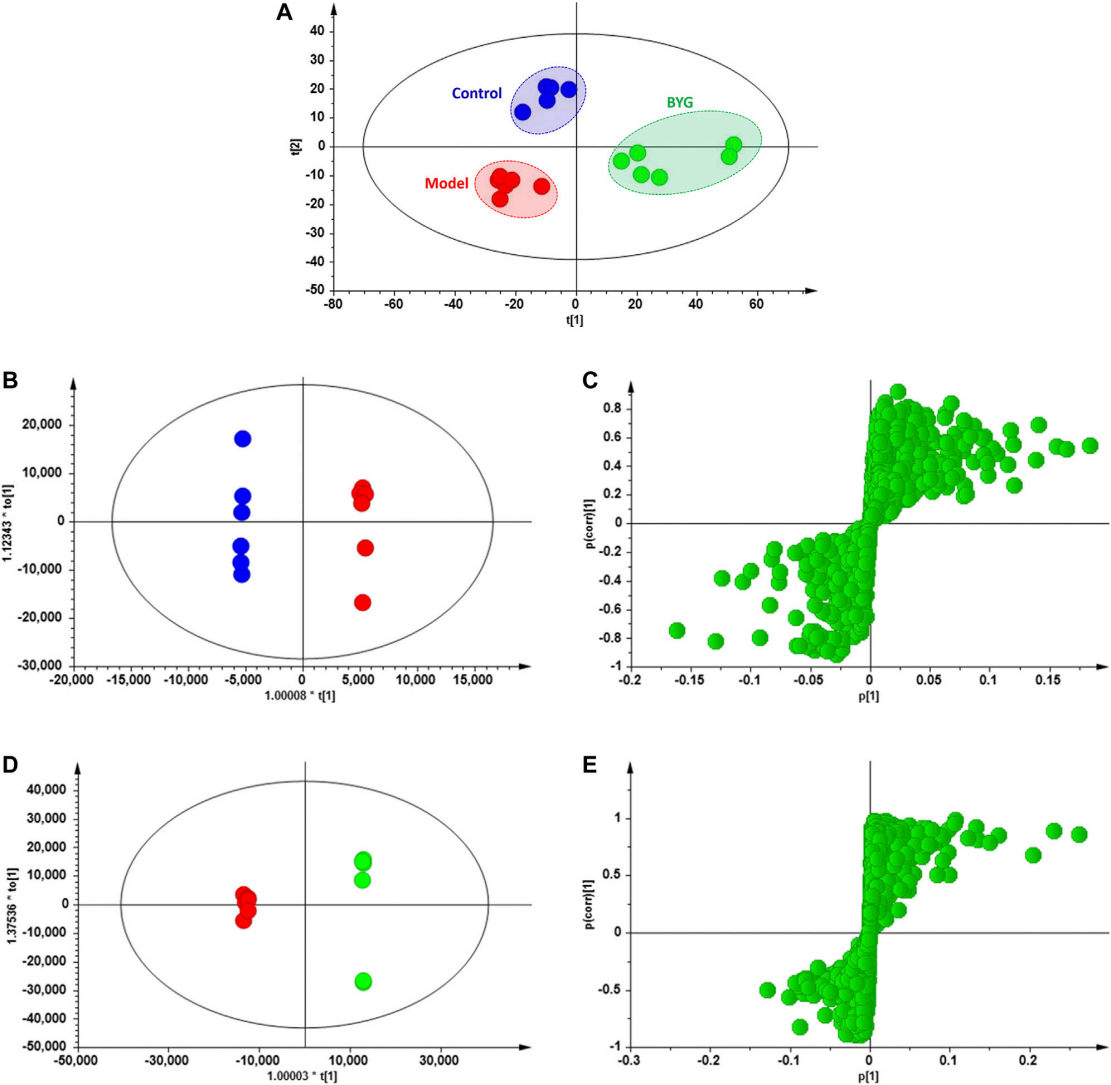
Scores plots of PLS-DA, OPLS-DA and S-plot analysis on the serum metabolic profiles of control (blue dot), model (red dot), BYG treatment (green dot) group.

As shown in Figure 7, the score plots from PLS-DA and OPLS-DA showed that the medical-induced incomplete abortion rat group was obviously separated from the pregnancy control group, which suggested that the administration of mifepristone and misoprostol could cause abnormalities of endogenous metabolites in rats. Moreover, compared with that of the model group, the metabolite profiles of the BYG group were gradually restored to normal.

**FIGURE 8:**
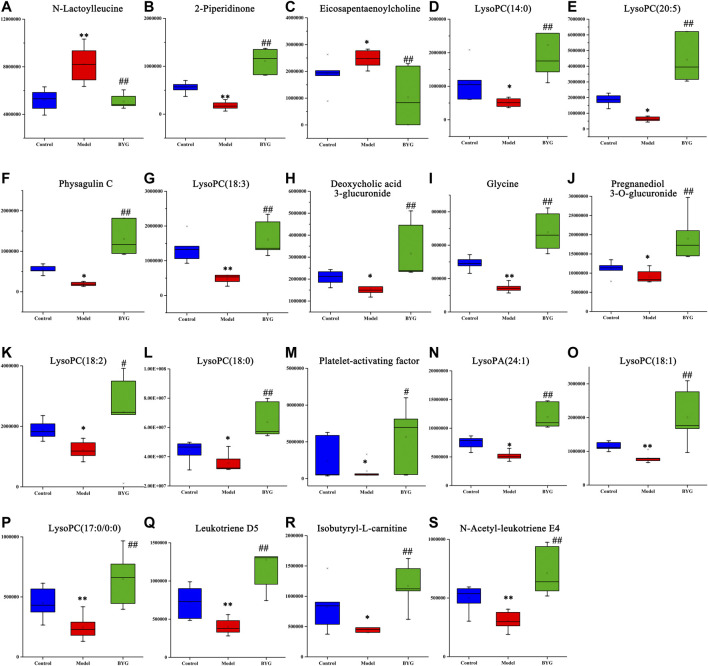
Boxplots of identical biomarkers normalized intensity of rat plasma. Note: VS control group, *p < 0.05 and **p < 0.01; VS model group, ^#^ *p* < 0.05 and ^##^ *p* < 0.01.

**FIGURE 9:**
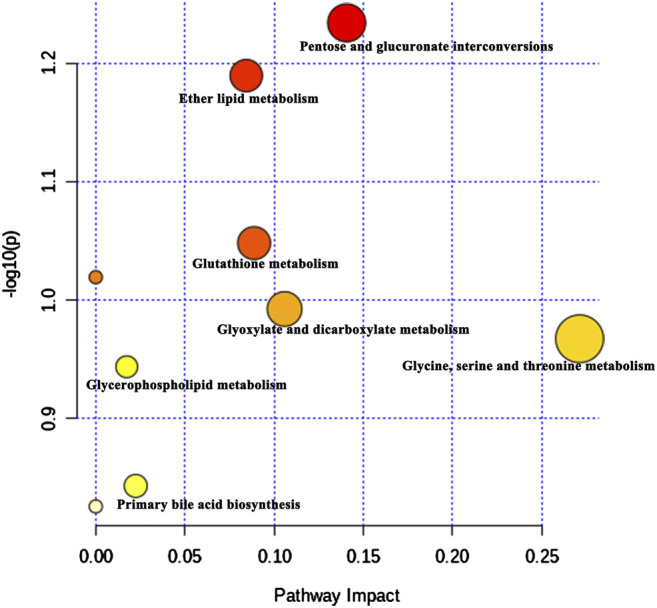
The potential metabolic pathways according to the identified biomarkers by MetaboAnalyst.

Furthermore, 19 kind of main endogenous biomarkers, including N-lactoylleucine, 2-piperidinone, isobutyryl-l-carnitine, eicosapentaenoylcholine, LysoPC(14:0), LysoPC(20:5), physagulin C, LysoPC(18:3), leukotriene D5, deoxycholic acid 3-glucuronide, glycine, pregnanediol 3-O-glucuronide, LysoPC(18:2), LysoPC(17:0/0:0), N-acetyl-leukotriene E4, LysoPC(18:0), platelet-activating factor, LysoPA(24:1), and LysoPC(18:1), which were mainly related to the amino acids metabolism, lipids metabolism and bile acid biosynthesis were identified according to the *m*/*z* results (Table 2). The peak area box diagram of each biomarker was shown in Figure 8. The analysis of potential metabolic pathways according to the identified biomarkers by MetaboAnalyst was shown in Figure 9. These results indicated that BYG exerts a potential protective role in the intervention of incomplete abortion by ameliorating the metabolic disorders.

**TABLE 2:** Identification results of main potential biomarkers changes.

Peak no.	R.T. (min)	Formula	Mass (*m*/*z*)	Biomarkers
1	1.02	C_9_H_17_NO_4_	204.1198	N-Lactoylleucine
2	1.76	C_5_H_9_NO	100.0758	2-Piperidinone
3	2.31	C_11_H_22_NO_4_	232.1538	Isobutyryl-l-carnitine
4	6.78	C_25_H_42_NO_2_	388.3860	Eicosapentaenoylcholine
5	9.55	C_29_H_41_NO_4_	468.3031	LysoPC(14:0)
6	9.60	C_28_H_48_NO_7_P	542.3176	LysoPC(20:5)
7	9.73	C_30_H_38_O_9_	543.3209	Physagulin C
8	9.80	C_26_H_48_NO_7_P	518.3189	LysoPC(18:3)
9	10.00	C_25_H_38_N_2_O_6_S	495.3220	Leukotriene D5
10	10.20	C_28_H_44_N_2_O_8_S	569.3338	Acid 3-glucuronide
11	10.78	C_28_H_52_NO_7_P	546.3472	Glycine
12	10.90	C_25_H_40_N_2_O_6_S	497.3346	Pregnanediol 3-O-glucuronide
13	11.07	C_26_H_50_NO_7_P	520.3303	LysoPC(18:2)
14	11.36	C_25_H_52_NO_7_P	510.3476	LysoPC(17:0/0:0)
15	11.89	C_25_H_39_NO_6_S	482.3185	N-Acetyl-leukotriene E4
16	12.54	C_26_H_54_NO_7_P	524.3638	LysoPC(18:0)
17	12.77	C_26_H_54_NO_7_P	524.4282	Platelet-activating factor
18	15.90	C_27_H_53_O_7_P	521.3343	LysoPA(24:1)
19	16.52	C_44_H_84_NO_8_P	786.5852	LysoPC(18:1)

## Discussion

Herba Leonuri, also named Chinese Motherwort, has been widely used for the treatment of dysfunctional uterine bleeding, dysmenorrhea, polycystic ovary syndrome and other gynecological diseases for hundreds of years (Lin et al., 2017; Lin et al., 2019). It has been reported that chemical components, including alkaloids, phenolic acids, flavonoids, as well as volatile oil and tannins, have been isolated and identified in Herba Leonuri (Wang et al., 2013; Wojtyniak et al., 2013). Alkaloids are considered to be the major bioactive ingredients of Herba Leonuri (Xie et al., 2015; Dong et al., 2017). Among these components, leonurine exhibits a variety of pharmacological activities, Research has confirmed that leonurine significantly reduces the IL-1β-induced production of NO, PGE2, IL-6, and TNF-α by inhibiting the activation of the PI3K/Akt/NF-κB signaling pathway (Yin and Lei, 2018).

*Angelica sinensis* (Oliv.) Diels has been considered to be “female’s ginseng” mainly because of its excellent therapeutic effect for gynecological diseases, such as dysmenorrhea, irregular menstruation, premenstrual, and menopausal symptoms (Huntley and Ernst, 2003; Haines et al., 2008; Hook, 2014). Ferulic acid, ligustilide and angelica polysaccharide are considered to be the main bioactive substances of Angelica (Wei and Huang 2015; Yang et al., 2019) Studies have confirmed that *Angelica sinensis* (Oliv.) Diels extract can be developed for the treatment of endometriosis by inhibiting inflammatory reactions (Xiong et al., 2020) and could significantly decrease NF-kB/β-actin and IL-6/β-actin mRNA expression in the uterus of rats with pelvic inflammation (Huang et al., 2012). In addition, metabolomics studies have shown that the volatile oil of *Angelica sinensis* can significantly inhibit the systemic inflammatory response caused by acute local stimulation, and mainly exert its anti-inflammatory activity by regulating glycine and arachidonic acid metabolic network disorders (Yao et al., 2015).

Previous studies have shown that endometrial damage and uterine tissue inflammation are the main pathological features of abnormal bleeding after abortion (Cicinelli et al., 2017; Care et al., 2018). Inflammatory cells increase in the endometrium because of the reduction in hormone levels after incomplete abortion (Evans et al., 2011). In this study, pathological examination revealed obvious edema and congestion in the uterus of the incomplete abortion rat model and BYG intervention had a certain regulatory effect. Based on our experimental results and previous studies, we speculate that Herba Leonuri and *Angelica sinensis* (Oliv.) Diels may be the main anti-inflammatory drugs in BYG.

Vascular endothelial growth factor (VEGF), which is closely related to angiogenesis, is considered to be a key factor in endometrial repair (Sugino et al., 2002; Maybin et al., 2011). Studies have shown that leonurine, the main active component of Herba Leonuri, can promote the angiogenesis of endothelial cells by activating the mTOR/ERK pathway and enhance angiogenesis both *in vivo* and *in vitro* (Wang et al., 2018). Previous results revealed that *Angelica sinensis* can promote angiogenesis and exert anti-apoptotic effects by activating of p38 MAPK/HIF-1αα/VEGF-A signaling (Cheng et al., 2017). The current study results showed that the expression of VEGFA in uterine tissue was decreased in the medical-induced incomplete abortion model group compared to that in the control group, and BYG treatment could increase the expression of VEGFA, indicating that BYG has a certain repair effect on endometrial injury after postpartum or abortion.

Glycerophospholipids are key components of the cells and are involved in metabolism and signaling. LysoPC(20:5) is lysophospholipid, which play an important role in lipid signal transduction by acting on the lysophosphatidic receptor (LPL-R) (Lingwood and Simons, 2010). Previous studies based on metabolomics have demonstrated that the serum NMR-based metabolomics analysis method was sensitive enough to distinguish individuals having artificial abortions from healthy individuals and can provide a further understanding of the mechanism of artificial abortion complications (Wu et al., 2018). An LC/MS based serum metabolomics study indicated that women with recurrent abortions have abnormal metabolism of purine, tyrosine and amino acids (Zhang et al., 2019). In a study on the Chinese traditional medicine prescriptions Taohong Siwu Docation’s intervention in the serum metabolic profile of rats with abnormal uterine bleeding, 23 biomarkers mainly involved in the metabolism of amino acids and lipids were identified (Zuo et al., 2019). Here we also observed a significant reduction in LysoPC(18:2), LysoPC(20:5), LysoPC(18:3), LysoPC(18:1), LysoPC(18:0), LysoPA(24:1) in the model group, which is consistent with the results of a previous study (Zuo et al., 2019).

N-Lactoylleucine is a lactoyl derivative of phenylalanine. An untargeted metabolomics screening study showed that the plasma levels of metabolites such as N-lactosyl-amino acids were closely correlated with the concentrations of lactate and amino acids (Jansen et al., 2015). The results of this study showed that there was abnormal amino acid metabolism in abortion rats, and the metabolite profiles of amino acids were gradually restored to normal with the treatment of BYG.

As demonstrated in the current study, in the model group, physagulin C and platelet-activating factor were significantly decreased compared to their levels in the control group, and BYG intervention could increase their levels. The most well-known pharmacological action of Angelica was enriching blood and invigorate circulation. Studies have indicated that *Angelica sinensis* can promote hematopoiesis and thrombopoiesis because of its anti-apoptotic activity through the PI3K/AKT pathway (Liu et al., 2010). Previous investigations suggested that α-pinene derivatives isolated from *Angelica sinensis* exhibited antithrombin and antiplatelet aggregation activity in vitro. (Yang et al., 2011). After administration of mifepristone and misoprostol, vaginal bleeding increased significantly in rats. The changes in these biomarkers may be closely related to the abnormal coagulation system, and BYG may reduce the amount of bleeding and shorten the bleeding time by regulating blood coagulation.

Progestational hormones, mainly progesterone, are secreted by luteal cells of the ovaries. The main metabolite of progesterone is pregnanediol. The urine excretion of pregnanediol can be used as an index to evaluate luteal function. Pregnanediol 3-O-glucuronide is a natural metabolite of pregnanediol produced by UDP glucuronosyltransferase in the liver. A metabolomics study has shown that pregnanediol 3-O-glucuronide level in placental metabolites of women that had spontaneous preterm births was significantly reduced (Elshenawy et al., 2020). Although we did not observe this effect of BYG on estradiol at the serum hormone level, based on the results of metabolomics, we speculated that BYG may also play a certain role in regulating the abnormal hormone levels postpartum or after an abortion.

## Conclusion

In summary, these results indicate that intragastric administration of mifepristone combined with misoprostol in early pregnancy could significantly disturb the metabolic profiles of rats. BYG exerts a potential protective role in the intervention of incomplete abortion by anti-inflammatory, promote endometrial repair, and regulate the metabolic disorders in the amino acids metabolism, lipids metabolism and bile acid biosynthesis.

## Data Availability Statement

The raw data supporting the conclusions of this article will be made available by the authors, without undue reservation.

## Ethics Statement

The animal study was reviewed and approved by Chengdu University.

## Author Contributions

LZ, J-YL, and YG conceived and designed the experiments. YZ, WL, T-TC, and YY performed the experiments. T-TC and YZ analyzed the data. YZ and M-YW wrote the paper.

## Funding

This work was supported by Sichuan Provincial Department of Education (17TD0010), the Youth Science Foundation of Chengdu University (2017XJZ15) and the Health and Family Planning Commission of Chengdu-Key disciplines of clinical pharmacy.

## Conflict of Interest

M-YW and YG were employed by the company Zhuzhou Qianjin pharmaceutical Co., Ltd.

The remaining authors declare that the research was conducted in the absence of any commercial or financial relationships that could be construed as a potential conflict of interest.
